# Urological surgery in the COVID-19 era: Patient counselling and informed consent

**DOI:** 10.1080/2090598X.2020.1772032

**Published:** 2020-05-25

**Authors:** Elsayed Desouky

**Affiliations:** a Department of Urology, Wexham Park NHS Hospital, Berkshire, UK; b Department of Urology, Alexandria Main University Hospital, Alexandria, Egypt

**Keywords:** COVID-19, pandemic, urologist, counselling, consent

## Abstract

The current coronavirus disease 2019 (COVID-19) pandemic is massively affecting our daily practice. Elective surgical service has been significantly altered, i.e. reduced overall service provision, special operating theatres’ precautions, as well as considerations for testing patients before surgery. The process of counselling patients and obtaining their consent is a must before any surgical intervention. Several factors can affect this process particularly amid the current pandemic crisis. Only with a full understanding of all the relevant facts, including risks and available alternatives, can patients give an ‘informed consent’. Therefore, we urologists need to be aware of the impact of the current COVID-19 situation on how to consent our patients.

**Abbreviations:**

COVID-19: coronavirus disease 2019; PPE: personal protective equipment

## Introduction

The coronavirus disease 2019 (COVID-19) pandemic has been a real challenge to healthcare systems and clinicians. We urologists are facing its impact every day in practice. Our clinical decision and service provision may need to deviate from the internationally accepted standard of care during this unprecedented situation. Consequently, surgical practice amid this pandemic is not at all simple or a straightforward process [1].

One of the essential pillars in our practice is getting the consent of our patients in order to proceed with any planned intervention. Mutual trust forms the foundation for a good relationship between the doctor and patient. Informed consent is a vital document while performing all surgical procedures, *especially* through this challenging time of the pandemic. Proper documentation and counselling of patients is important in any informed consent [2].

Patient counselling is not a process of giving advice, but it is a process of helping patients who are genuinely in need. It is a professional communication to help a patient in making a decision. Counselling is different from a casual conversation, as it builds a professional relationship with the patient. It is totally focussed, specific and purposeful [2].

Scientific aspects are not the only key factors in decision-making, as the patients choice is significantly affected by factors such as age, beliefs, sexual health, level of education, and cognitive state [3]. This is particularly significant in an unprecedented time like the current pandemic crisis. Our clinical decisions may deviate from the standards into other alternatives, different patients can decide on different treatment modalities even when falling into the same risk group.

During such a pandemic it is understandable that individuals feel emotional unrest. The healthcare professionals, particularly the surgeon, should take this into consideration while counselling patients before surgery. The Urologist should provide adequate aid and guidance for individuals with existing health conditions, as well as those experiencing enhanced emotional distress during the COVID-19 outbreak.

When counselling patients, come to eye level with them and talk calmly and clearly. Reassure patients that you want to minimise any discomfort or concerns they may have about the care they are receiving. Take time to ask about and listen to patients’ most significant concerns. Although there may not be clear answers or solutions, the surgeon needs to display openness and honesty. Reflect back what you have heard the patient say and identify the emotion the patient is communicating [4].

Non-verbal expression is an integral part of effective communication while counselling the patient [2], so urologists need to acknowledge the challenges to effective communication presented by personal protective equipment (PPE), i.e. masks, face shields, and other barriers that limit non-verbal expression. This can cause additional distress to patients, as they are not familiar with such looks of their doctors especially amid the current pandemic crisis.

From an infection-control point of view, patient counselling and obtaining an informed consent can pose a risk of infection, as it can involve challenging the rules of social distancing. Keeping a 2-m distance between the surgeon and the patient may not be easy to follow given such clinical circumstances. In addition, COVID-19 statuses are variable among those who need urological surgery, from asymptomatic patients to those admitted into COVID-19 wards or even in intensive care units. Accordingly, different levels of PPE have been suggested depending on the hazard of exposure to COVID-19 (Table 1) [5].

**Table 1.** Different levels of protection using PPE amid the COVID-19 pandemic [5].

Level	COVID-19 risk	PPE
1	Low risk Asymptomatic	Gloves Apron Surgical mask Surgical cap Visor/goggles/glasses Surgical scrubs
2	COVID-19 + ve or high risk (symptoms + fever) Low aerosol risk	Gloves Surgical scrubs Gown Surgical mask Surgical cap Visor/goggles/glasses
3	COVID-19 + ve or high risk Receiving aerosol generating treatment (e.g. mechanical ventilation, CPAP, high pressure nasal oxygen) in ICU/ITU/HDU	Gloves Surgical scrubs Gown FFP3 mask (fitted) or ventilated hood Surgical cap Visor/goggles/glasses

CPAP: continuous positive airway pressure; FFP: filtering facepiece; HDU: High-Dependency Unit; ICU: Intensive Care Unit; PPE: personal protective equipment; ITU, Intensive Therapy Unit.

The process of informed consent entails both ethical and legal aspects. The ethical side emphasises the patient’s ‘autonomy’ and basics of human rights. The patient is the only one to decide what should/should not happen to his/her body. Nobody else is to decide for the patient or coerce him/her to make a particular decision. Even the doctor can only act as a facilitator in the decision-making process. The legal side arises from the fact that no one has the right to touch, let alone treat, another person without permission otherwise it is a physical assault and is punishable [2].

For the patient’s consent to be ‘informed’, the surgeon must truthfully disclose accurate, adequate and relevant information in a form and language that the patient can understand. Consent cannot be a patient’s signature obtained routinely by a staff member [2].

Currently, COVID-19 is posing an enormous pressure on all healthcare systems, leading to reductions in elective surgical service provision. This needs to be taken into consideration, as it will definitely impact the process of getting an ‘informed’ consent from our patients.

As per COVID-19 recommendations by European Association of Urology (EAU) guidelines panels, there are some recommendations to be considered and discussed with patients during counselling (Table 2) [6].

**Table 2.** Recommendations for urological surgery amid COVID-19 [6].

**General recommendations for surgical procedures**
Depending on the resources and capacity they recommend treating only high-priority and emergency cases surgically during the COVID-19 pandemic. Consider not only equipment, OR and ICU beds capacity but also blood supplies available, drugs shortage in order to prioritise your surgeries. Consider that even if capacity is available low priority patients increase the footfall and the risk of COVID-19 transmission between patients and staff. Consider that surgery has been reported to be harmful in asymptomatic patients who subsequently tested COVID-19 positive. Consider treating intermediate priority patients if capacity is available but not during the COVID-19 surge. Consider older patients with comorbidity at severe risk of COVID-19 infection and a fatal outcome. Therefore, carefully balance if in high-priority cases surgery is the only alternative. Where ventilator capacity for COVID-19 patients has been breached, high-priority surgical candidates requiring ICU ventilation should be triaged according to local recommendations – or if unavailable – age and comorbidity.
**Surgical considerations for known COVID-19-positive patients (and suspected cases)**
A specially equipped dedicated OR has to be prepared for these cases. For endourology, a mobile C-arm fluoroscopic X-ray system for radiological imaging and experienced personal for its handling has to be in the special OR. Surgeons and operating team in OR should be completely protected against infection of COVID-19 and adopt adequate protection devices. All minimally invasive procedures should be preferably performed by experienced surgeons and with the minimum number of experienced OR staff members required. Additionally, no external observer is allowed in the OR. To date, there is no specific data demonstrating an aerosol presence of the COVID-19 virus released during minimally invasive abdominal surgery. It is recommended lowering electrocautery power setting as much as possible in order to reduce the surgical smoke production especially in laparoscopic surgery. Evacuation of irrigation fluid during endourological procedures (cystoscopy, TURB, BPH endoscopic surgery, URS, RIRS, PCNL) should be collected through a close system.
**Testing patients before surgery in the time of COVID-19**
Patients with clinical symptoms like fever and respiratory distress should all undergo preoperative COVID-19 test. In an emergency situation it is suggested to handle those patients as a COVID-19-positive patient in order to reduce risk of contagion. Patients without any clinic symptoms and without travel history to endemic areas and previous contact in the last 2 weeks with a COVID-19-positive patient: Testing of elective patients is recommended whenever possible within 48 h prior to surgery in an outpatient clinic setting. Strongly recommend for patients to comply with general directions regarding social distancing as stated by the government since this will likely lower the risk for COVID-19 disease at the time of operation.

ICU: Intensive Care Unit; OR: Operating room; PCNL: percutaneous nephrolithotomy RIRS: retrograde intrarenal surgery; TURB: transurethral resection of bladder; URS: ureterorenoscopy.

The Royal College of Surgeons (RCS) England guidelines suggest: the risk to the surgical patient should be a combined assessment/discussion of the real risk of proceeding under current circumstances, versus the real risk of delay. Any available alternative treatments must be discussed [1].

The BAUS recently published some important recommendations to consider upon consenting patients for surgical intervention during the COVID-19 pandemic, which can vary depending on the surgery being an emergency or semi-urgent [5]:

- Confirm that the patient has not had COVID-19 symptoms in the last week.
- Confirm that no member of their household is currently unwell with COVID-19 symptoms.
- The patient may need to be admitted to hospital a day or two before surgery.
- A chest CT scan may be required before surgery and, if suggestive of COVID-19 infection, this may result in surgery being cancelled.
- Coming into hospital may increase the chance of contracting COVID-19, or the patient may already be carrying it when presenting for the operation.
- The patient may be tested for COVID-19 prior to surgery and the result may not be available before proceeding in emergency cases.
- Swab testing has a sensitivity of only ~75% (66–80%). A single negative swab test does not exclude COVID-19 (especially if obtained from a nasopharyngeal source alone, or if taken relatively early in the disease course).
- The true outcomes of COVID-19 infection in surgical patients are not currently well known.
- The patient’s care may be delayed, performed differently or cancelled at short notice during the COVID-19 pandemic.
- If the patient contracts, or develops, COVID-19 whilst in hospital, recovery from surgery could be made more difficult with increased chances of serious illness or death.
- Should the patient require emergency intensive care support, there is no guarantee that it will be available.
- Should the patient wish to delay surgery, we are unable to provide a realistic time frame as to when it could next be scheduled.
- It cannot be guaranteed that a particular surgeon will perform the procedure but that the operation will be performed by an appropriately trained specialist.
- There is a higher than normal chance that patients will be moved to another ward/hospital during their postoperative recovery.
- Visitors may not be allowed during the patient’s stay in hospital.
- Care may be on mixed wards where other patients may not have been tested for COVID-19 status.

If patients are not able to make decisions for themselves, the urologist must work with those close to the patient and with other members of the healthcare team. The urologist must take into account any views or preferences expressed by the patient and must follow the law on decision-making when a patient lacks capacity [1].

Additionally, Urologists must respect a patient’s decision to refuse an investigation or treatment, even if we think their decision is wrong or irrational. We should explain our concerns clearly to the patient and outline the possible consequences of their decision but we must not, however, put pressure on a patient to accept our advice [1].

Urologists need to explain to the patients all about the perioperative circumstances that might affect the outcome of their planned surgery, *particularly* those related to COVID-19 situation, so as to help them in making their own decision.

## Conclusion

The COVID-19 pandemic imposes a novel approach to patient counselling and obtaining consent from our patients. Not only do urologists need to explain the details of the surgical procedure itself, but also to fully disclose to our patients the impact of the current pandemic situation on service provision and how this may affect them. This is essential in order to get a proper ‘informed’ consent in keeping with good medical practice.
